# From Glucose to Neuroprotection: Exploring Antidiabetic Medications as a Novel Approach to Alzheimer's Disease Treatment

**DOI:** 10.7759/cureus.70710

**Published:** 2024-10-02

**Authors:** Chandaraa Kumar Pandiyan, Arjun Gokulan Manivannan, Narayanan Jaishankar, Chitra Vellapandian

**Affiliations:** 1 Department of Pharmacy/Pharmacology, Sri Ramaswamy Memorial College of Pharmacy, Sri Ramaswamy Memorial Institute of Science and Technology, Kattankulathur, IND

**Keywords:** alzheimer's disease, antidiabetic drugs, diabetes mellitus type 2, insulin, metformin

## Abstract

Alzheimer's disease (AD) is a major neurological disorder and a leading cause of dementia, which is characterized by progressive cognitive decline. Emerging research highlights the link between AD and type 2 diabetes mellitus (T2DM), suggesting that shared pathophysiological mechanisms, such as insulin resistance, inflammation, and oxidative stress, contribute to both conditions. This connection has led to the concept of type 3 diabetes. Given these overlaps, antidiabetic drugs are being explored for repurposing as potential AD treatments. Intranasal insulin, metformin, thiazolidinediones, and GLP-1 analogs like liraglutide have shown promise in reducing amyloid-beta levels and inflammation, and improving cognitive functions. Despite encouraging preclinical and early clinical results, challenges remain in translating these findings into safe and effective treatments. Continued research could lead to innovative therapies that address both AD and T2DM, offering improved patient outcomes.

## Introduction and background

The devastating neurological disease known as Alzheimer's disease (AD) affects countless people throughout the world, making it a major public health concern in the 21st century. AD, the primary cause of dementia, is marked by a progressive decline in cognitive abilities such as reasoning, memory, and the capacity to carry out daily activities. AD is primarily driven by the accumulation of beta-amyloid plaques and tubulin-associated unit (tau) tangles in the brain, leading to neuronal damage and cognitive decline. This disrupts synaptic communication, triggers inflammation, and eventually causes neuronal death. Biomarkers, such as brain imaging of amyloid beta (Aβ) plaques, extracellular Aβ peptide deposits, and intracellular deposits mostly consisting of hyperphosphorylated microtubule-associated protein tau, are known as neurofibrillary tangles (NFTs). With this pathology, the clinical signs of AD can be augmented by assessing abnormalities in brain volume and quantifying tau and Aβ levels in the cerebrospinal fluid (CSF) for dementia diagnosis. The condition usually initiates with slight memory loss and disorientation but gradually hampers an individual's capacity to interact, make judgments, and identify close family members. AD often occurs alongside other medical conditions, known as comorbidities, which can complicate both diagnosis and management. These comorbidities can exacerbate symptoms, affect the progression of AD, and impact the overall quality of life for patients. An increasing body of research suggests that having type 2 diabetes mellitus (T2DM) raises the probability of developing AD. Hyperglycemia and dysregulation of insulin action may contribute to amyloid plaque formation and neurodegeneration. The chronic metabolic disorder diabetes mellitus (DM) has emerged as a global health issue, impacting millions of adults globally. Projections suggest a substantial rise in the next few decades. This difficult condition is defined by the presence of chronic hyperglycemia, which describes unusually elevated blood glucose levels brought on by impairments in either the action or production of insulin. There are two primary forms of diabetes: type 1 and type 2. The immune system attacks and damages the pancreatic beta cells that produce insulin in type 1 autoimmune disease and type 2, which is mainly caused by insulin resistance and is typically linked to obesity and lifestyle factors [1].

**Figure 1:**
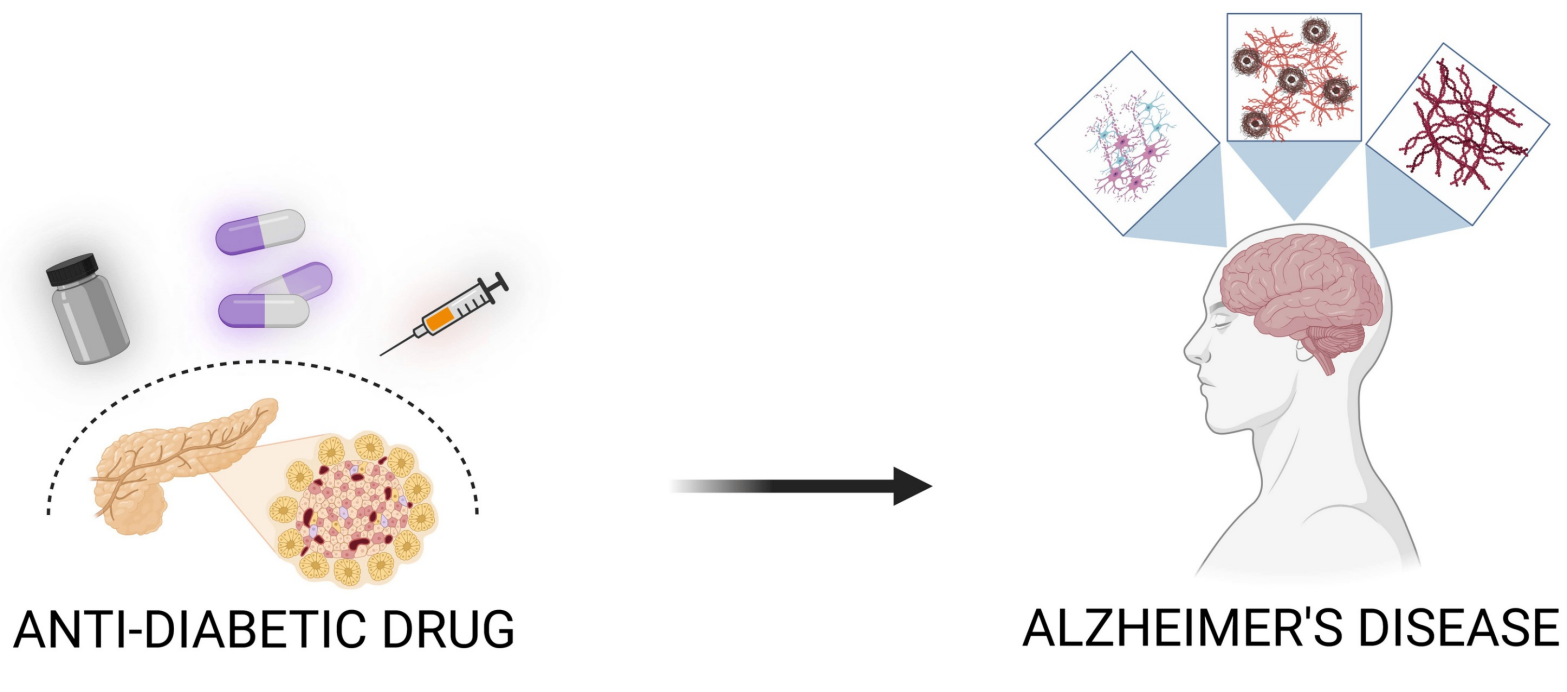
Antidiabetic drugs used for the treatment of AD AD: Alzheimer's disease Image credits: The image was illustrated by the author Chandaraa Kumar Pandiyan

In recent years, diabetes, particularly T2DM, has been realized as a significant potential danger for gradual brain changes, seen by biochemical, cellular, and structural changes, impairment of brain function, and ultimately an elevated risk of dementia [2]. Initially, it was believed that the brain was an organ incapable of responding to insulin. Nevertheless, the organ is currently recognized as insulin-sensitive due to an increasing amount of proven data in neurodegenerative studies. The developing knowledge of the brain's responsiveness to insulin has provided insight into the possible connection between the disorders associated with insulin and dementia [3]. Numerous epidemiological studies show a strong correlation between cognitive impairment and diabetes, particularly T2DM, caused by the inability of neurons to absorb glucose for energy generation effectively. The complex interplay between T2DM and AD encompasses various factors such as insulin resistance, inflammation, oxidative stress (OS), glutamate kinase 3β, insulin growth factor signaling, Aβ production from amyloid precursor protein (APP), NFT formation, and acetylcholine esterase activity regulation due to common pathways across type 1 diabetes mellitus (T1DM), T2DM, and AD. Although the anomalies observed in AD exhibit similarities with T1DM and T2DM, they are still characterized by the simultaneous occurrence of deficits in tropic factors and resistance to tropic factor receptors. Researchers have coined the term type 3 diabetes. Type 3 diabetes is a proposed term that refers to the potential link between diabetes and AD. It suggests that insulin resistance and impaired glucose metabolism in the brain contribute to the neurodegenerative processes seen in AD, leading some researchers to describe AD as type 3 diabetes. This concept highlights the role of insulin dysregulation in cognitive decline. The review paper examines the common cellular and molecular links between diabetes and AD in classifying type 3 diabetes [4]. This review includes the common cellular and molecular pathogenesis and mechanism of action for T2DM and AD; in this paper, we highlight the possibility of repurposing antidiabetic drugs as a strategy for Alzheimer's therapy by analyzing current market available drugs and clinical trials as well as integrating with their moa. Figure 1 shows the antidiabetic drugs used to treat AD.

## Review

Epidemiology

World prevalence rates of diabetes (T1 and T2) and AD have surged dramatically in recent decades, reflecting broader demographic and lifestyle changes. According to the World Health Organization (WHO) report, rising obesity rates, poor diets, and physical inactivity are the main causes of diabetes, which affects over 500 million people worldwide [5]. According to the Centers for Disease Control and Prevention, modern lifestyle factors and an aging population are strongly associated with T2DM, which is responsible for roughly 90%-95% of these cases [6]. According to the International Diabetes Federation Atlas, T1DM, although less common and typically diagnosed in childhood or early adulthood, makes up the remaining percentage. In contrast, among dementias, AD is the most popular and is also increasing, largely due to longer life expectancies and an aging global population [7]. AD accounts for 60%-70% of the approximately 55 million individuals who are currently living with dementia, according to the WHO's most recent estimates. The National Institute of Aging reports that the prevalence of AD dramatically increases with age, affecting approximately 5%-8% of individuals aged 65 and older and increasing to 25%-50% among those aged 85 and later [8]. Projections of the Alzheimer’s Association suggest that by 2050, the number of Alzheimer's cases could nearly triple, underscoring a growing global health concern. Both diabetes and AD illustrate significant challenges driven by contemporary lifestyle and demographic trends, emphasizing the need for effective prevention and management strategies [9].

The common pathway of AD and diabetes

**Figure 2:**
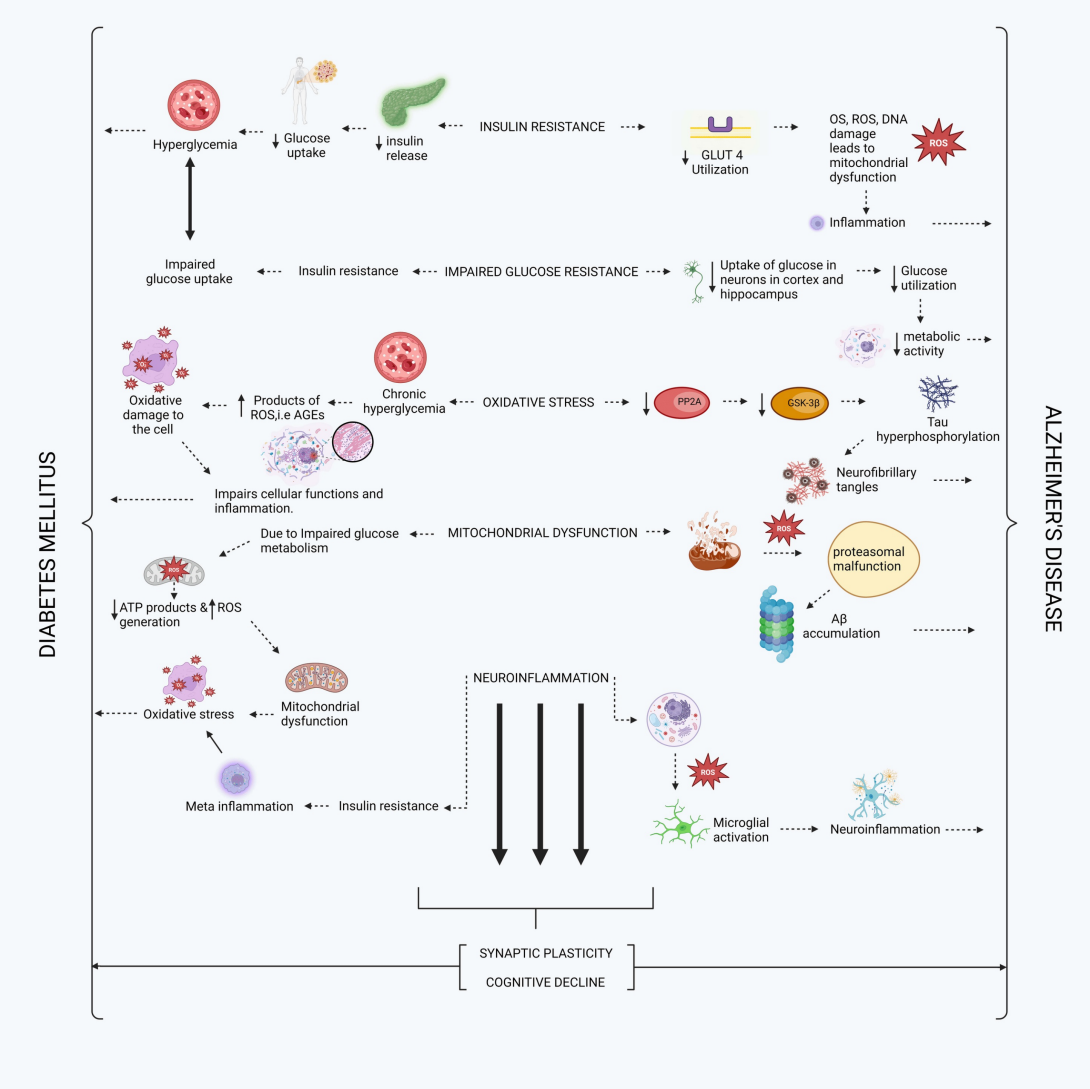
Commonly shared pathology of Alzheimer's and diabetes GLUT4: glucose transporter 4; OS: oxidative stress; ROS: reactive oxygen species; DNA: deoxyribonucleic acid; AGEs: advanced glycation end products; PP2A: protein phosphatase 2A; GSK-3β: glycogen synthase kinase-3 beta; ATP: adenosine triphosphate; Aβ: amyloid beta Image credits: The image was illustrated by the author Chandaraa Kumar Pandiyan

An overview of diverse and similar pathophysiology underlying the causes of DM (T1DM and T2DM) and AD includes insulin resistance, poor glucose resistance, mitochondrial dysfunction, OS, neuroinflammation, synaptic plasticity, and cognitive decline. Insulin resistance, impaired glucose resistance, mitochondrial dysfunction, OS, and neuroinflammation contribute to the emergence of two further types of pathology called synaptic plasticity and cognitive decline. Insulin resistance reduces insulin release, results in low glucose uptake, and leads to hyperglycemia, which, in turn, causes DM. Conversely, it reduces the utilization of glucose transporter type 4 receptors, leading to OS, reactive oxygen species (ROS), and DNA damage. This, in turn, leads to mitochondrial dysfunction, which further induces inflammation and ultimately causes AD. Impaired glucose resistance inhibits insulin release, resulting in impaired glucose uptake, hyperglycemia, and, ultimately, DM. Conversely, a lack of glucose uptake by neurons in the brain's cortex and hippocampus due to poor glucose resistance leads to inadequate glucose utilization. This, in turn, reduces metabolic activity and leads to AD. In OS, characterized by chronic hyperglycemia, the products of ROS advanced glycation end products lead to oxidative damage in the cell, thereby impairing its function. Inflammation, on the other hand, ultimately results in DM. Additionally, the reduction of protein phosphatase 2A and glycogen synthase kinase-3 beta enzymes in the cell leads to tau hyperphosphorylation, which, in turn, causes NFTs that cause AD. ROS affects cells during mitochondrial dysfunction, leading to proteasomal malfunction. The buildup of Aβ protein will lead to AD, as well as problems with glucose metabolism, less adenosine triphosphate (ATP) products, and more ROS. This will make mitochondrial dysfunction happen, which will lead to OS and, finally, diabetes. When a cell experiences neuroinflammation due to ROS, it triggers the activation of microglia, leading to further neuroinflammation and the development of Alzheimer's. Conversely, this resistance to insulin results in metainflammatory and OS within the body, ultimately leading to diabetes. Finally, as mentioned in the above paragraph, the combined consequences of insulin resistance, impaired glucose resistance, mitochondrial dysfunction, OS, and neuroinflammation are responsible for developing two additional forms of pathology known as synaptic plasticity and cognitive decline. AD and diabetes ultimately come to an end. The pathway between DM and AD is shown in Figure 2.

Current DM drugs used for the treatment of AD

Intranasal Insulin

An established risk factor for developing Alzheimer's is DM-insulin resistance. Studying the impact of insulin on our bodies will provide a clear therapeutic approach, and insulin reduces Aβ levels in our body system. Hypoglycemia is a known adverse side effect of peripheral insulin injection. Nevertheless, intranasal insulin does not influence blood sugar levels; it reaches the brain through olfactory neurons. Insulin exhibits neuroprotective and antioxidant properties, among its ability to reduce Aβ and misfolded hyperphosphorylated tau (p-tau) levels. Using intranasal insulin, a different study by Appleby and Cummings found that people with Alzheimer's and Alzheimer's melancholy (aMCI) had better cognitive function, lower tau/a-beta 1-42 ratios, and less hypometabolism on brain fludeoxyglucose positron-emission tomography (PET) scans, which are symptoms of AD [10].

Sulfonylureas

An older class of antidiabetic medications called sulfonylureas (SUs) works by interfering with the pancreatic ATP-sensitive potassium (KATP) channels to increase insulin production. It is interesting to note that neurons also have KATP channels. In db/db mice, glipalamide therapy decreased hippocampus Aβ, prevented neuronal death, and improved hippocampal synaptic plasticity, according to a recent scientific study by Boccardi et al. It revealed that taking SU in addition to metformin reduced the risk of Alzheimer's by 35% [11].

Metformin

Metformin has recently garnered considerable scholarly attention in the treatment of AD. In the mouse model of AD, metformin improves cognition and decreases tau phosphorylation and Aβ pathogenic markers. In rats, low-dose metformin has been observed to lessen OS and scopolamine-induced cognitive impairment significantly. Metformin treatment also enhances memory and learning capacity in the senescence-accelerated mouse model by lowering APPc99 and p-tau levels [12].

Metformin is a biguanide medication approved for the treatment of T2DM. Contradictory findings have been derived from empirical observations of this medication in AD models. Metformin upregulated β-secretase 1 to enhance the formation of Aβ species when given alone. However, when metformin was given with insulin, the amount of Aβ was decreased. Metformin reverted the neuropathological and molecular characteristics when provided to an insulin-resistant neuron model of AD [13].

Thiazolidinediones Glitazones

Pioglitazone and rosiglitazone are peroxisome proliferator-activated receptor (PPAR)-γ agonists that aid in managing T2DM. They are also being studied for their potential in treating AD. Research conducted in vitro has shown that the combination of rosiglitazone and insulin has synergistic neuroprotective effects, while rosiglitazone treatment alone results in partial neuroprotection. Numerous studies using animal models of AD have shown that rosiglitazone therapy reduces inflammation, Aβ oligomers, and cognitive impairment. The effects of rosiglitazone on glucose regulation are not correlated with its anti-AD properties. With varying degrees of success, several randomized controlled trials have examined rosiglitazone's impact on individuals with AD and amnestic mild cognitive impairment (aMCI). From the study by Appleby et al., the treatment group's cognition improved without affecting plasma Aβ levels, while in another, the cognition of non-apolipoprotein E (APOE)-4 carriers improved. Even when humans were categorized according to the APOE-4 allele status, these outcomes were not replicated in further clinical trials. Although thiazolidinediones are effective in the treatment of AD, rosiglitazone has been reported to have notable adverse effects, including stroke, myocardial infarction, and edema. In 2010, rosiglitazone use for the treatment of T2DM was banned in the United States and Europe due to its side effects. PPAR agonists appear to alleviate several aspects of AD pathophysiology in animal models, such as decreased expression of inflammatory genes, decreased levels of amyloid, and neuroprotective effects, which are associated with calcium homeostasis in cultured hippocampal brain neurons [13].

The second therapeutic agent, pioglitazone, has a place in both animal models and randomized controlled trials [13]. According to this research, pioglitazone can reduce inflammation and OS while restoring normal cerebral blood flow (CBF). Pioglitazone administration has been observed to improve cognition in aMCI and AD patients with associated T2DM. Reductions in tumor necrosis factor-α (TNFα) levels, enhanced CBF, and elevated Aβ(1-40/1-42) ratios were also linked to improved cognitive performance in treatment groups [14].

GLP-1 Analogs

An experimental study has examined the relationship between AD and two glucagon-like peptide-1 (GLP-1) analogs. The treatment involves the prescription of liganduvide for T2DM, primarily via modulating insulin levels in reaction to increasing fasting blood glucose levels. A laboratory experiment utilizing liraglutide treatment demonstrated neuroprotective efficacy and increased long-term potentiation. The results relating reduced cerebral Aβ oligomers, dentate gyrus neurogenesis, and anti-inflammatory activity were the same in another study that used animal models of AD. An in vitro study on exenatide revealed its neuroprotective properties and capacity to correct tau hyperphosphorylation. From the study by Appleby et al., it has been proven in an animal model that lixisenatide and liraglutide can cross the blood-brain barrier and stimulate the dentate gyrus to produce new neurons [13].

Liraglutide is a man-made version of GLP-1 that is quite similar, with a 97% similarity. This medication exerts multiple effects on AD by penetrating the blood-brain barrier. These effects include diminishing the presence of Aβ plaques, mitigating insulin receptor and synaptic abnormalities, and reducing cognitive impairments. Synaptic plasticity serves as a connection in the brain that links T2DM and AD. Studies have demonstrated that liraglutide enhances spatial learning and memory and protects against synaptic plasticity deficits in the CA1 region of the hippocampus. Liraglutide not only has preventative characteristics but also is able to prevent the progression of AD in mouse models by reversing a number of key pathological features. Incretins and incretin analogs have a neuroprotective impact on the brain, as demonstrated by the ability of long-acting liraglutide to restore the distribution of insulin receptors on cell membranes in a rat model with AD (APPSWE/PS1dE9), hence alleviating insulin signaling abnormalities.

Furthermore, the methodical use of liraglutide in transgenic mice with AD for eight weeks effectively halted the neurodegenerative consequences often observed in AD. These include neuronal depletion, cognitive decline, and reduced synaptic plasticity. It showed that these substances improve the growth of cells, promote memory and synaptic plasticity, and decrease the presence of amyloid plaques, OS, and inflammation [14].

Recent drugs in clinical trials for AD

Amylin Analogy (Pramlintide)

Amylin is secreted alongside insulin by the pancreatic beta cells in response to glucose consumption. Amylin, found in the pancreas, controls insulin and glucagon release. It helps regulate blood glucose levels and decreases the rate of gastric emptying. Oligomerization of the human amylin hormone can impair its functionality. Amylin oligomerization and deposition frequently occur in individuals with obesity and prediabetic insulin resistance who exhibit elevated hormone secretion. More than 95% of individuals with T2DM exhibit amylin amyloid buildup in the pancreatic islets, which is considered harmful to the cells and reduces flexibility. Amylin and Aβ may have comparable pathophysiology in the brain. This theory is proposed based on the observation that amylin and Aβ can create harmful oligomers and amyloid fibrils. Therefore, pramlintide, an analog of amylin, has just been authorized to manage T2DM. Its purpose is to avoid the occurrence of hyperamylasemia and the subsequent release of oligomerized amylin into the bloodstream. This may perhaps function as an innovative therapeutic focus for the management of diabetic brain injury and AD. The preclinical evidence of AD animal models showed that the administration of the amylin analog pramlintide reduced OS and neuroinflammation while also improving memory. There is currently no accessible data on the use of this molecule for the treatment of AD in humans. Additionally, there are no ongoing clinical trials involving this molecule for AD treatment [11].

NE3107-HE3286

In a number of preclinical illness models, including rheumatoid arthritis, chronic obstructive pulmonary disease, DM, and Parkinson's disease, the pharmaceutical substance NE3107 demonstrated anti-inflammatory properties. Reduced release of inflammatory cytokines such as TNFα, interferon-gamma, interleukin-1α, and transforming growth factor beta is the result of NE3107's suppression of the nuclear factor kappa B (NF-κB)/extracellular signal-regulated kinase (ERK) pathway. Blocking the NF-κB/ERK pathway decreases OS, glial activation, and the production of Aβ and p-tau. NE3107 hinders the activation of mitogen-activated protein kinases, which play a role in the regulation of insulin resistance, leading to a decline in hyperinsulinemia and hyperglycemia. Since it is currently an ongoing study, the outcomes of this investigation may confirm the ability of anti-inflammatory insulin sensitizers to reduce cognitive deterioration, enhance function in individuals with AD, and decrease disease progression [15].

Semaglutide

Semaglutide is a drug that acts as an agonist for GLP-1. It has been specifically designed to treat individuals with obesity as well as T2DM. Epidemiological research findings suggest that those diagnosed with diabetes who undergo semaglutide therapy have a reduced likelihood of developing dementia in comparison to those who are prescribed alternative antidiabetic drugs. It is believed that semaglutide's anti-inflammatory characteristics, which it achieves by reducing inflammation in the peripheral tissues, are the primary therapeutic benefit in AD. Semaglutide has limited or negligible penetration of the blood-brain barrier, with only minimal quantities reaching specific regions of the brain [15]. Semaglutide is also a peptide-based drug that activates the GLP-1 receptors and can potentially control AD accumulation. GLP-1 receptors play a role in synaptic transmission, cognition in hippocampus neurons, and cell apoptosis. Therefore, they can be targeted for research on potential neuroprotective and cognitive-enhancing medicines [16]. Semaglutide, a GLP-1 receptor agonist, is being investigated in a research study titled EVOKE Plus (NCT04777409) for its potential therapeutic effects in individuals with early AD. Participant recruitment for this study is ongoing and aims to explore whether semaglutide, commonly used for managing T2DM, can offer benefits in slowing cognitive decline in patients with early-stage AD by targeting neuroinflammatory pathways and enhancing neuronal survival. Semaglutide is formulated in a pharmaceutical composition that includes sustained-release microspheres containing either the analog itself or a pharmaceutically acceptable salt. This formulation is the subject of a research study titled EVOKE (NCT04777396). A phase 3 clinical trial (NCT04777396) of semaglutide was initiated in May 2021 to assess the effectiveness of semaglutide in patients with early AD, which is currently recruiting participants to investigate its potential therapeutic effects in individuals with early AD. The study aims to assess whether the sustained-release formulation of semaglutide can provide neuroprotective benefits and slow cognitive decline in early-stage AD patients. The main aim of this research is to evaluate the shifts in the Clinical Dementia Rating Scale Sum of Boxes score from the beginning of the research to week 104, and since research is currently ongoing, the results have not been reported [17].

Apolipoprotein E

Obicetrapib, also known as TA 8995, is a compound. Obicetrapib is a medication that inhibits cholesteryl ester transfer protein and is used to reduce levels of low-density lipoprotein when taken with a statin drug. Clinical trials for obicetrapib are currently underway for a variety of disorders, including diabetes, dyslipidemia, atherosclerosis, AD, and heterozygous familial hypercholesterolemia. Epidemiologic studies have shown promise for statins as a potential risk factor for AD. Also, according to some research, cholesterol may increase the degeneration of nerve cells in this disorder. These findings provide the groundwork for future research into the possible therapeutic benefit of obicetrapib in AD patients [18]. Under the clinical trial registration number NCT05161715, obicetrapib is now participating in a phase 2 open-label exploratory proof-of-concept study. Participants in this study who are adolescents and have a confirmed clinical diagnosis of AD supported by biomarker data showing abnormalities in tau and amyloid biomarkers, as well as neurodegeneration, will be treated. A patient needs to have at least one APOE4 gene copy. Over 24 weeks, participants will be closely observed by the researchers. The primary endpoints of the study will be the concentrations of apolipoproteins in both plasma and CSF. Additionally, the concentrations of high-density lipoprotein particles in both plasma and CSF will be assessed. As the study is still underway, the results are yet to be released [15].

Metformin (Glucophage)

Metformin is a pharmacological agent that enhances insulin sensitivity, consequently resulting in a decrease in blood glucose levels. Furthermore, it has been demonstrated to possess anti-inflammatory characteristics, reduce OSs, and promote the growth of new neurons in preclinical studies. The biguanide medicine metformin decreases glucose production in the liver, decreases intestinal glucose absorption, and increases insulin sensitivity in specific tissues, leading to lower blood glucose levels. Metformin effectively reduces overall mortality, cardiovascular disease, and cancer and also treats T2DM [15].

Metformin is now being tested in a clinical experiment in phase 2/3. The trial comprises 370 individuals who have received a diagnosis of amnestic low cognitive impairment (MCI). The trial is registered with the identifier NCT04098666. Subjects will be randomly assigned to either receive medication or a placebo for 24 months. The main metric being assessed is the free and cued selective reminding test. Given the ongoing status of the study, outcomes remain unpublished [17].

Insulin

Insulin exerts several effects on the brain and has several potential therapeutic benefits in AD. Neurons with Alzheimer's exhibit insulin resistance, although insulin has a positive impact on bioenergetics and brain metabolism. Furthermore, insulin has been found to have various other functions in tissue investigations and animal models of AD. These include influencing synaptic plasticity, the creation of dendritic spines, the turnover of transmitters, proteostasis (including the removal of Aβ and p-tau), inflammation vascular function, and lipid metabolism [18]. The results of the trials conducted until now have predominantly been unfavorable. However, new trials are currently investigating innovative methods of distribution and trial designs [19,20]. Insulin, a biologic of considerable size, necessitates administration by subcutaneous, intravenous, or nasal inhalation routes. Creating efficient distribution methods for clinical studies, including people with Alzheimer's, has proven difficult. An investigation will be conducted on a group of 40 persons who are cognitively normal but have tested positive for amyloid. This research aims to determine how feasible it is to use a device that administers insulin nasally (NCT05006599). The principal aim of this work is to ascertain the ratio of prescribed doses that participants successfully consumed. A new clinical study will investigate the safety and effectiveness of combining insulin with a sodium-glucose cotransporter type 2 inhibitor called empagliflozin (NCT05081219). After amyloid PET or CSF AD biomarkers have confirmed 60 participants as amyloid positive, we will observe them for eight weeks. These participants will either have cognitively normal or mild AD dementia, moderate cognitive impairment (MCI), or AD. Evaluating the treatment's safety and tracking the incidence of any new or major events that occur throughout the treatment are the primary goals of this study. They are both ongoing phase 2 clinical trials, and the results have not yet been published [17].

Discussion

The investigation of antidiabetic drugs for the management of AD is both promising and intricate. The convergence of pathophysiological pathways in AD and T2DM, including insulin resistance, inflammation, OS, and impaired glucose metabolism, establishes a robust basis for studying the repurposing potential of both medications.

One of the most studied approaches is the use of intranasal insulin. This method bypasses systemic circulation and directly targets the brain, which is particularly important since insulin resistance in the brain is a key feature of Alzheimer's pathology. Intranasal insulin has shown neuroprotective effects, such as lowering Aβ levels and reducing tau phosphorylation, which are central to AD development. Importantly, this method does not cause hypoglycemia, making it a safer option for AD patients. Clinical studies have demonstrated improvements in cognitive functions among patients treated with intranasal insulin, suggesting its potential as an AD therapeutic. Metformin, another widely used antidiabetic drug, has garnered attention for its potential cognitive benefits. Preclinical studies indicate that metformin may reduce AD-related biomarkers, such as amyloid plaques and NFTs. Metformin's ability to decrease OS and inflammation, both implicated in AD, further supports its exploration as a treatment option. However, the results have been mixed. While some studies report cognitive benefits, others have not found significant improvements when metformin is used alone, highlighting the need for further investigation. Thiazolidinediones, such as rosiglitazone and pioglitazone, have also been studied in AD. These drugs improve insulin sensitivity and have shown varying degrees of effectiveness in clinical trials. Rosiglitazone initially showed promise in reducing inflammation and slowing cognitive decline in AD patients. However, due to its adverse cardiovascular effects, it was eventually withdrawn from the market. Pioglitazone, on the other hand, has shown potential in reducing inflammation and improving CBF, particularly in diabetic patients with AD. However, its effects on nondiabetic AD patients have been less conclusive, warranting further research. GLP-1 analogs, such as liraglutide and lixisenatide, are another class of antidiabetic drugs with potential benefits for AD. These drugs have shown promise in preclinical studies by enhancing neurogenesis, reducing amyloid plaques, and improving cognitive functions. Their ability to cross the blood-brain barrier and exert neuroprotective effects makes them attractive candidates for AD research. Ongoing clinical trials of newer antidiabetic agents, like pramlintide and NE3107, reflect the growing interest in exploring innovative therapeutic strategies for AD. Pramlintide may help mitigate amylin-related brain damage, while NE3107's anti-inflammatory properties offer the potential for treating AD.

Despite the encouraging preclinical and early clinical results, several challenges remain. Translating these findings into effective and safe human treatments requires rigorous clinical trials. Factors such as drug delivery mechanisms, potential side effects, and patient-specific responses must be carefully evaluated. Overall, the investigation of antidiabetic drugs in the context of AD reflects a promising and innovative approach to addressing both conditions. As research advances, these drugs may offer new therapeutic options that harness the shared pathophysiological pathways between T2DM and AD, potentially leading to more effective and holistic treatment strategies.

## Conclusions

In conclusion, repurposing antidiabetic drugs for AD represents a novel approach that shows great promise. Both AD and T2DM have several pathophysiological features, according to the available evidence, such as insulin resistance and OS, prompting the type 3 diabetes concept. Research shows that various antidiabetic drugs, including insulin analogs, metformin, SUs, thiazolidinediones, and GLP-1 analogs, may offer neuroprotective benefits and cognitive improvements in AD. Intranasal insulin, metformin, and GLP-1 analogs like liraglutide and semaglutide have shown promise in reducing Aβ levels and inflammation. Ongoing trials of amylin analogs and anti-inflammatory agents further support this potential. While results are still emerging, these drugs hold substantial promise for advancing Alzheimer’s treatment. Continued exploration of this link could pave the way for innovative treatments that address both diseases simultaneously, ultimately improving patient outcomes and quality of life.
